# Nonrandomized Trial of Feasibility and Acceptability of Strategies for Promotion of Soapy Water as a Handwashing Agent in Rural Bangladesh

**DOI:** 10.4269/ajtmh.16-0304

**Published:** 2017-02-08

**Authors:** Sania Ashraf, Fosiul A. Nizame, Mahfuza Islam, Notan C. Dutta, Dalia Yeasmin, Sadika Akhter, Jaynal Abedin, Peter J. Winch, Pavani K. Ram, Leanne Unicomb, Elli Leontsini, Stephen P. Luby

**Affiliations:** 1 Department of International Health, Johns Hopkins Bloomberg School of Public Health, Baltimore, Maryland.; 2 Enteric and Respiratory Disease Program, Environmental Intervention Unit, Infectious Disease Division, International Centre for Diarrheal Disease Research, Bangladesh (icddr,b), Dhaka, Bangladesh.; 3 Department of Epidemiology and Environmental Health, School of Public Health and Health Professions, University at Buffalo, Buffalo, New York.; 4 Division of Infectious Diseases and Geographic Medicine, Stanford University, Stanford, California.

## Abstract

We conducted a nonrandomized trial of strategies to promote soapy water for handwashing in rural Bangladesh and measured uptake. We enrolled households with children < 3 years for three progressively intensive study arms: promotion of soapy water (*N* = 120), soapy water promotion plus handwashing stations (*N* = 103), and soapy water promotion, stations plus detergent refills (*N* = 90); we also enrolled control households (*N* = 72). Our handwashing stations included tap-fitted buckets and soapy water bottles. Community promoters visited households and held community meetings to demonstrate soapy water preparation and promote handwashing at key times. Field workers measured uptake 4 months later. In-depth interviews and focus group discussions assessed factors associated with uptake. More households had soapy water at the handwashing place in progressively intensive arms: 18% (promotion), 60% (promotion plus station), and 71% (promotion, station with refills). Compared with the promotion-only arm, more households that received stations had soapy water at the primary handwashing station (44%, *P* ≤ 0.001; 71%, *P* < 0.001 with station plus detergent refill). Qualitative findings highlighted several dimensions that affected use: contextual (shared courtyard), psychosocial (perceived value), and technology dimensions (ease of use, convenience). Soapy water may increase habitual handwashing by addressing barriers of cost and availability of handwashing agents near water sources. Further research should inform optimal strategies to scale-up soapy water as a handwashing agent to study health impact.

## Introduction

Diarrhea and pneumonia remain leading causes of death in children < 5 years mainly in low-income countries.^1^,^2^ Handwashing with soap can reduce the incidence of diarrhea and acute respiratory illnesses in children < 5 years in addition to other infectious diseases such as soil-transmitted helminthiasis and trachoma.^3^–^7^ Repeated episodes of diarrhea are associated with increased risk of pneumonia and malnutrition.^8^ Malnutrition can have long-term consequences such as stunting, cognitive decline, and impairment of human productivity.^9^

Handwashing with soap is effective in reducing hand contamination and is recommended after fecal contact and before eating and handling food.^4^,^10^,^11^ Washing hands with soap at these key times is associated with reduced risk for childhood diarrhea.^4^,^12^ Despite large-scale campaigns to promote handwashing with soap, actual practice among low-income communities is low especially after fecal contact (∼19%) and before handling food (< 1%).^13^–^16^ Structured observations conducted in 11 countries found that on average only 17% of caretakers wash hands with soap after fecal contact.^17^ Although 90% of respondents in rural Bangladesh, knew critical handwashing times, few in fact used soap before handling food or after defecation.^18^ In rural Bangladesh, handwashing agents range from soil, ash to bar or liquid soap, where rates of soil or ash use after fecal contact are comparable to soap use.^19^ Relatively high costs of bar soap (US$0.45–0.55) and reluctance to leave soap in convenient public places, due to concerns of theft or wastage by children, are barriers to handwashing with soap in low-income communities.^20^,^21^ Observational studies support that having a convenient place to wash hands that has soap and water are associated with higher rates of handwashing after fecal contact compared with those who did not.^22^–^24^

Formative research conducted before this feasibility trial had tested multiple culturally appropriate handwashing devices in both rural and urban settings.^25^ Results had suggested that soapy water, a water solution of powdered laundry detergent, could be an acceptable handwashing agent in resource-poor communities. Soapy water has been promoted in low-income countries including Kenya,^26^,^27^ Bangladesh,^28^,^29^ and Peru.^30^ In a noninferiority trial, the microbiological efficacy of soapy water was similar to bar soap for removing fecal indicator bacteria during handwashing in urban Bangladesh.^10^

We aimed to test low-cost alternatives to bar soap for handwashing in rural Bangladesh. We assessed relative feasibility and acceptability of alternative strategies for promotion of soapy water as a handwashing agent. The goal of this trial was to inform intervention design of a larger randomized controlled trial in rural Bangladesh looking at the impact of water, sanitation, hygiene (WASH) and nutrition interventions called WASH Benefits.^31^

## Materials and Methods

### Setting.

This study was conducted in Kishoreganj, a district in central Bangladesh. We selected a subdistrict, Karimganj, which had no known ongoing water, sanitation, and hygiene interventions during the study period. Within subdistricts, the smallest unit of administration is a union comprising several villages. Within villages, households are set up as compounds, typically with clusters of four to seven households generally surrounding a common courtyard.

### Design.

The study was a nonrandomized trial, with allocation to intervention intensity at the village level. We purposively selected four proximate unions, which were closest to the main road for ease of implementation. From a list of the total number of villages in these unions, we chose three proximate villages closest to the major roads for each intervention. Formal meetings and information sessions were held with local government officials and community leaders at both the union and the village level for overall permission to proceed. Field workers identified the center of each of these villages and went door to door to identify households that had a child aged < 3 years. Field workers selected compounds with at least two households with children in that age group, and sought written informed consent from the adults in the compound and from the guardian of each child for enrollment into the study. Each intervention arm was implemented in three villages. We enrolled 15 compounds from each village. Every enrolled compound in the same village received the same intervention.

We set the sample size, a total of 60 compounds from sets of three villages each, to allow development and piloting of the behavior change intervention strategy for the promotion of handwashing with soapy water. We based the size on logistical limits rather than a power calculation and predicted effect sizes.

### Intervention.

The intervention arms were designed to gain an understanding of whether and how the uptake improved by addressing hardware-related barriers with provision of a handwashing station or monetary constraints by providing free detergent to make soapy water instead of using more expensive bar soap. We tested three progressively intensive packages: 1) promotion of soapy water only, 2) promotion of soapy water with a handwashing station, and 3) promotion of soapy water with project-provided handwashing stations and free detergent refills. We compared each intervention arm against a control arm where we did not provide any intervention.

The key theoretical constructs guiding intervention components were derived from the integrated behavioral model for water, sanitation, and hygiene (IBM-WASH), an ecological framework that draws from several theories including a combination of the health belief model and the social cognitive theory.^32^ The intervention targeted two key handwashing times, both of which have been shown to be associated with reductions in childhood diarrhea in rural Bangladesh: 1) after fecal contact and 2) before food preparation.^33^ Fecal contact events included toilet use, cleaning a child's anus after defecation, and feces disposal. Handwashing during food preparation included washing hands before contact with food that would not be cooked further. The targeted group was the primary caregiver of children < 3 years of age and other family members in the household. The intervention was developed by a multidisciplinary team of anthropologists, sociologists, and trained field workers used by the International Center for Diarrheal Disease Research, Bangladesh (icddr, b) and implemented over 9 months from October 2010 to June 2011.

**Figure 1.**
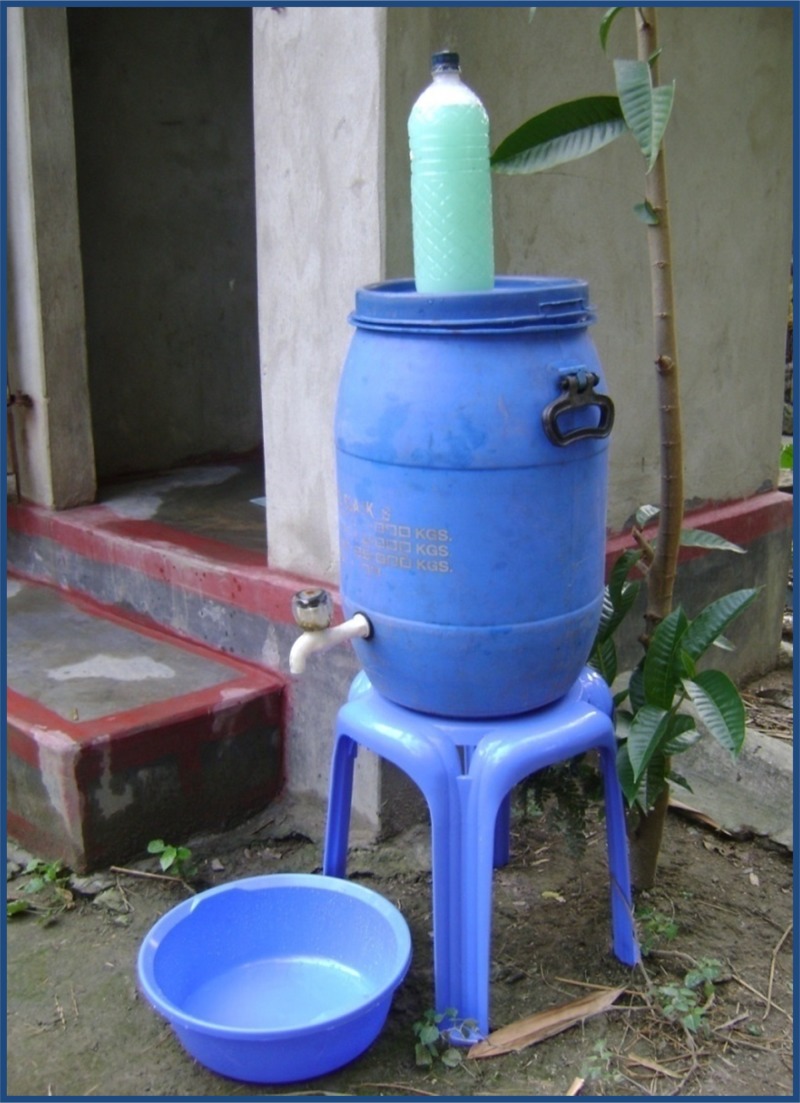
Handwashing station and soapy water bottle distributed to trial households in rural Kishoreganj, Bangladesh, 2011.

Soapy water was a mixture of detergent (30 g; the amount supplied in widely, commercially available sachets) and water (1.5 L) contained in a plastic bottle, with a hole on its top, so that a user can squeeze the soapy water onto their hands when needed. We provided a 1.5-L plastic bottle for caregivers to make and store soapy water (Figure 1

). Our handwashing stations (wholesale cost per unit US$6.5) included a 40-L plastic bucket with tap and lid, a bowl as a hand basin and a stool to place the bucket on.^25^ This enabling technology was recommended after formative research conducted in similar rural communities that highlighted the need for water storage capacity, and durability. Convenient placement of the station contributed to the perceived ease of the use of enabling technologies for handwashing.^25^

A local female community member was trained as a health promoter to conduct interactive household visits and courtyard sessions using live demonstrations, flip charts, and cue cards for each arm. The promoter was required to have at least 12 years of formal education and was paid US$13 (1,000 taka) per month as a stipend for their contribution. They worked approximately 48 hours, reflecting the hourly rate for a day laborer, the most common source of earned income in Bangladeshi rural communities. Promoters were trained to assist the field team in delivering hardware and consumables where applicable, negotiate placement of hardware, answer questions and make recommendations regarding use, and promote habit formation using messages developed by the research team. The messages included personal and social benefits of handwashing, self-efficacy to make and use soapy water. Flip charts were revised through community feedback to aid promoters to communicate the importance of hygiene, highlighting both health benefits such as reducing childhood diarrhea and nonhealth benefits such as being good parents and neighbors by protecting their children's health and having better smelling hands. Promoters placed cue cards showing how to prepare and use soapy water near latrines and the kitchen area to prompt handwashing. These messages were disseminated uniformly across all arms.

In all the arms, promoters visited households twice a week and conducted one courtyard session per month for 4 months. In the promotion-only arm, no enabling technology was provided to the households. The promoter showed them how to make soapy water using detergent available in their household or identified those available in the closest market place. She encouraged them to place separate soapy water bottles near their latrines or kitchen. In the arm that received enabling hardware plus promotion, two sets of handwashing stations and soapy water bottles were provided per eligible household, one to be located near the toilet and other at the food preparation area. In the third arm, bimonthly detergent refills were delivered in addition to the two sets of handwashing stations and soapy water bottles. A control arm was also enrolled to assess for contextual changes during the study; these compounds did not receive any intervention.

### Data collection.

At baseline, before any promoter visits, trained field workers asked the primary caregiver questions on demographic characteristics, household possessions, and self-reported handwashing behaviors, and where they most often washed their hands; they also observed the availability of water and hand cleansing agents at that place. They measured the distance of this handwashing place, in steps, from the toilet and the kitchen, and asked the mother and a child < 3 years to show their hands for visual inspection of general cleanliness; they asked the caregiver to demonstrate how they usually washed their hands after defecation. Responses were recorded on handheld computers. We conducted a baseline survey at enrollment and a follow-up survey 4 months after the start of the intervention in each arm. The primary quantitative outcome for handwashing uptake was the proportion of households with observed soap or soapy water together with water at a dedicated handwashing place.

**Figure 2.**
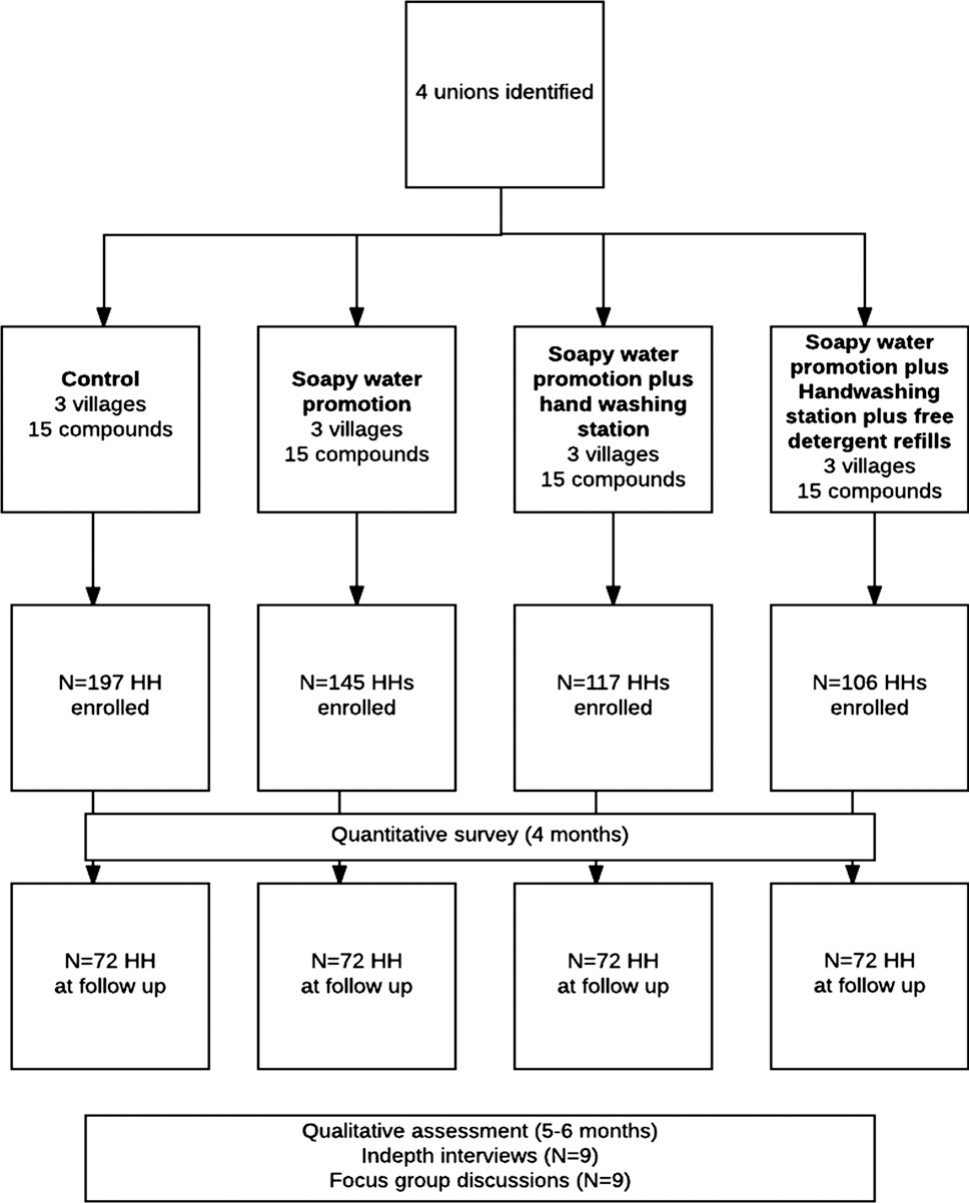
Study population and assessment profile, Kishoreganj, Bangladesh, 2010.

In March and April 2011, 5–6 months after the start of the intervention, four qualitative researchers conducted in-depth interviews (*N* = 9), and group discussions (*N* = 9) with primary caregivers in Bengali, the local language. One respondent from each village in the three intervention arms (total *N* = 9) was selected in consultation with the local promoter for the in-depth interviews. Six female care givers and three male household heads were interviewed. Primary female caregivers for the focus group discussions were chosen from those who had not already been interviewed. Interviews were conducted to understand user experiences with the handwashing technologies, motivations, and barriers to washing hands. Focus group discussions were used to discuss features of the technologies, suggested improvements, and common themes of the user experience and motivators to continue use. Participants were chosen to reflect the general demographic characteristics of the target population, such that the female respondents were housewives and the males worked locally as farmers or small businesses. All had less than 5 years of primary education. The researchers took voice recordings and detailed field notes and debriefed after both interviews and focus group discussions. Interviews were conducted in a private setting, mostly in the household of the study participants. Data saturation was used to determine the total number of interviews and group discussions.^34^ The intervention continued for up to a total of 8 months to document implementation lessons, logistic challenges to aid context-specific behavior change materials revision to improve soapy water intervention activities (Figure 2

). We continued collecting qualitative data until the end of the intervention to inform the final intervention design and recommendations going forward.

### Data analysis.

We summarized key household characteristics across the intervention arms at baseline. We analyzed the impact of intervention arms based on our primary handwashing-related outcomes of interest including proportions of households with soap and/or soapy together with water at a handwashing place, and visual observations of hand cleanliness noting any visible dirt on the palm or fingers of the hands.

To explore the impact of additional enabling technologies on soapy water uptake, we compared change in uptake indicators across groups. We compared the change in indicators for the arm that received 1) only soapy water promotion, 2) promotion plus provision of a handwashing station, and 3) promotion, provision of a handwashing station, and detergent refills. To evaluate whether provision of detergent led to a significant change in uptake we compared those who received the station (2) with those to received station plus detergent (3). The difference-in-difference (DiD) estimate is the difference between the two differences, providing a measurement of the additional uptake in each arm. We used Stata version 10 to estimate DiD and associated *P* values using generalized linear regression, with robust standard errors adjusting for clustering at the compound level.^35^ For the DiD estimation, we used an interaction term to determine the difference in proportions of the key indicators at baseline across groups (1 versus 2, 1 versus 3, and 2 versus 3) and the difference in key indicators in each arm from baseline to follow-up.

All qualitative interview and group discussion transcripts were coded for themes drawn from IBM-WASH by native Bengali-speaking researchers, and the coded data were translated into English. The data were then manually analyzed and summarized according to the themes.

### Ethical considerations.

Consents from local governments and village representatives were also taken before enrolling eligible households. All households provided written informed consent. The protocol was reviewed and approved by human subjects review committees at icddr, b.

## Results

In total, 131 compounds in 12 villages were screened to identify 839 households in compounds with at least two children aged less than 3 years. Of these, 197 households assigned to the control arm, 120 to the promotion-only arm, 103 to the promotion plus hardware arm, and 90 to the hardware plus refills arm consented to participate and were retained for analysis in this study. The control arm initially enrolled 197 households, where the aim was not to intervene with any hardware or promotion except for data collection at two time points. Of these, 100 households were enrolled in a subsequent hygiene pilot intervention study leaving 97 households for this study that had no intervention at the 4-month uptake assessment (Table 1). Of these control households, 72 consented for the end line survey. The households remaining in the control group were not different from those excluded in terms of demographic characteristics: average age of primary caregiver was 30 years and had low education rates (42% none, 41% primary education). We did not observe significant changes in any of the measured indicators from the larger group at baseline to the reduced group at endline (Table 2). Household baseline demographics, facilities, and assets were similar across intervention arms (Table 1); 40% of the mothers had some formal education. The average household consisted of six members. Outdoor handwashing stations near the tube well or the water source were predominant ranging from 72% in the promotion plus handwashing arm to 92% in the promotion-only arm (Table 1). The proportion of households at baseline who had any soap near the handwashing station was low and differed across arms, with only 4.4% in promotion plus handwashing station arm compared with 18% in the handwashing station plus refills arm (Table 1).

### Comparative uptake of handwashing interventions.

**Figure 3.**
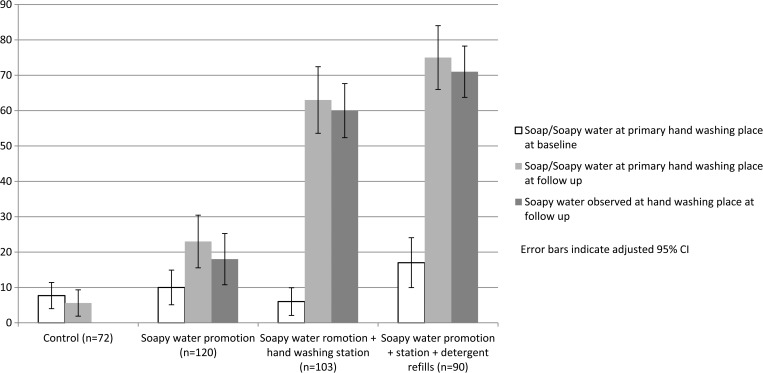
Presence of soap and soapy water at the handwashing place after 4 months of intervention, Kishoreganj, Bangladesh, 2010.

At unannounced 4-month follow-up visits, soap or soapy water were observed together at the handwashing place in 6% of control, 23% of promotion-only, 63% of handwashing station plus bottles, and 75% of stations plus detergent households (Figure 3

). Proportions of households with observed soap and water together was significantly higher in all intervention arms (*P* < 0.05) than that observed at baseline and compared with the control arm. At follow-up, soapy water bottles were observed at handwashing stations in 24/120 (20%) households from promotion-only arm, 82/120 (79%) from hardware, and 77/90 (86%) households from hardware plus detergent refill arm (Figure 3). When asked to demonstrate usual handwashing behavior after defecation, significantly more respondents in all intervention arms washed their hands with soap or soapy water compared with baseline. Visual observations of hand cleanliness of the mothers and children under 3 years did not differ across the arms at follow-up (Table 2).

Soap or soapy water were more likely to be observed at a primary handwashing place at follow-up from baseline in arms that received free handwashing stations with soapy water bottles (DiD: 47%, *P* < 0.001) or with detergent refills (48%, *P* < 0.001) compared with the arm with promotion only (Table 3). Provision of detergent did not lead to significantly greater soap/soapy water availability at handwashing stations compared with those who received hardware.

### Qualitative results.

We organized the factors affecting use of soapy water using the multilevel framework of IBM-WASH.^32^ We identified factors at the individual, household, and the community level spanning the contextual, psychosocial, and technology dimensions.

### Convenience and ease of use.

#### Psychosocial and technology dimensions at the individual and household levels.

Participants from the arms who received a handwashing station reported that the station helped them to store water at locations convenient to the toilet and the kitchen, which facilitated handwashing. Before the interventions, the primary caregivers reported that they only washed hands with soap near the tube well after defecation. The availability of soapy water near a water source prompted them to wash their hands after defecation as well as after handling feces. One mother who received a station said, “Now hand washing is easy for us as soapy water and water is ready together.” A caregiver (35 years old) explained “I have no tube well in my house. After receiving the hand washing station we could use it as a water reservoir along with the soapy water bottle at the toilet. So children could easily use it after defecation.”

Participants reported that the handwashing station and the soapy water bottle was visible especially near the kitchen and worked as a reminder to wash hands. A 29-year-old caregiver said “We remember to wash hands when we see the hand washing station. The provided hand washing station made hand washing easy for us. I have installed the drum and soapy water near the toilet and all of my family members can wash their hands just near the toilet which is easy for them.”

There were a few complaints about leakages from handwashing stations, specifically where the tap joined the drum. These were reported through the community promoters and defect stations were replaced. Manufacturers were notified to double check the assembly to avoid these recalls.

### Perceived value and sharing.

#### Contextual, psychosocial, and technology factors at the household and community level.

Participants were less concerned about the soapy water (bottle) being stolen making it easier to leave in public spaces for sharing. They also mentioned that soapy water was less expensive compared with regular bar soap and was very easy to make. In trial arms where detergent was not provided, participants from some low-income households reported hesitation to buy detergent solely for the purpose of making soapy water. Some parents reported that children misused the soapy water and used water in the handwashing stations for purposes other than handwashing.

Because of the perceived value of the provided handwashing station, household members expressed concern regarding theft during the night. This led some households to keep the stations indoors after dark hours, making it unavailable during the night to facilitate handwashing. A 30-year-old caregiver stated “I always look after my hand washing station similar to other possessions such as my television, mobile phone etc. I keep my hand washing station near the toilet in the morning and move it inside my room at night because of the possibility of theft during the night, as the toilet is so far from my room.”

### Household roles.

Mothers took the primary responsibility of promoting good handwashing practice among their family members by encouraging their children, husbands, and in-laws. Mothers reported that they taught their children how to wash hands themselves and often also assisted with the process, especially for younger children who could not independently wash their hands. Usually mothers maintained the handwashing station by refilling it when empty. Adult male household members noted that they could not use the handwashing station during the day when they worked outside the home.

The community lost interest in the facilitated courtyard meetings after the first two or three sessions. They reported that listening to the same issues repeatedly was tiring but they welcomed new discussion topics when they were introduced. These information sessions, persuasion by the health promoters, and presence of the handwashing station with soapy water were identified by household members as key factors for encouraging washing hands with soapy water.

## Discussion

Soapy water was an acceptable and popular hand cleansing agent, especially when provided with a handwashing station and a bottle in rural Bangladesh. Relatively low cost, ease of preparation, and willingness to locate the bottle near the water source in place of traditional bar soap contributed to its acceptability. Our uptake findings are consistent with other studies promoting soapy water usage in Kenya^26^,^36^ and Bangladesh.^28^ These findings can be understood using the multilevel framework of IBM-WASH.^32^ Various dimensions such as contextual (shared compound setting, socioeconomic status), psychosocial factors (existing norms, knowledge), and technology factors (design, cost, and ease of use) affected the behavior of washing hands with soapy water. Studies have found that theory-based infrastructure for handwashing that meets the target population's needs achieve better uptake.^39^ Hulland and others emphasized the features of a handwashing station that was acceptable in the rural Bangladesh context.^25^ Our findings extend this work by measuring uptake of soapy water with or without the handwashing stations. More households had soap and water together with the provision of enabling handwashing stations and detergent refills. We found factors at the community, household, and individual levels affected the acceptability of the handwashing station. At the community and household level, placement of the handwashing station at convenient locations affected use and sharing of product. Soapy water bottles were observed near a water source such as the tube well or the provided handwashing station. Although we did not specifically measure intervention uptake among those not included in the promotional activities, neighbors of intervention compounds were observed using soapy water during the qualitative assessment. Households valued handwashing stations, and many chose to move these indoors after dark to prevent theft. This perceived value could be a strength of the technology design that improved its use and maintenance at the household or individual level.^32^

Provision of a handwashing station increased accessibility of water and soap together at the same place (85/103 [83%] at follow-up compared with 9/98 [6%] at baseline). Soapy water bottles were observed in 79% (82/103) of households in the hardware plus promotion arm. This highlights the additive benefit of enabling technology that effectively addressed environmental barriers to handwashing by providing soap and running water together. In rural Bangladesh, soap is usually kept inside the house and not near the latrine or the kitchen or the water source where they might need to wash their hands at critical times.^19^ Interventions such as those tested in this pilot increased the presence of soap and water at a designated place that can improve handwashing behavior especially after fecal contact.^24^,^37^ In the intervention arm where a handwashing station was not provided, the soapy water bottle was placed near a water source, most commonly the tube well to wash and rinse hands. The presence of a station may have also increased handwashing during critical times, such as during food preparation in the kitchen area which may be distant from a water source. Providing the station made it easy for participants to try out the intervention, and judge if they like it without having to purchase it. It is conceivable that future interventions might not need to provide stations to all households, but to a subset to demonstrate their utility and encourage uptake.

We saw a significant increase in the availability of soap/soapy water by providing enabling technology compared with promotion-only, but no significant additional gain was recorded by providing free detergent (Table 3). This highlights the value users placed on the system and their willingness to keep it supplied, without ongoing product provision by program implementers. Soapy water was adopted by 20% of households who were offered only handwashing promotion messages. Promoter-led household and community meetings were effective in encouraging households to make their own soapy water. However, household members reported that these promotional activities became unattractive to attend over time and would benefit from revisions to minimize monotony. This level of interpersonal communication is expensive to maintain and deliver effectively over extended periods. Delivering strategic demonstrations using social networks can be used to stimulate behavior change.^38^ Tactical demonstration and use by community and opinion leaders may be successful when implemented with less frequent promoter-led efforts. Research to inform low-cost approaches will be valuable to inform more sophisticated approaches that have lower per person cost for longer term intervention. Recent studies have demonstrated that environmental nudges could improve handwashing in Bangladeshi children in resource-poor setting.^40^

There are several limitations in this study. The small scale and short duration of this pilot study does not allow us to project long-term uptake rates or health impact of soapy water in rural low-income settings, though we did have 4 months of follow-up, which provides insights on practices beyond the immediate intervention “honeymoon” period. Findings from this pilot study were used to revise and improve the design of a customized handwashing station, details of which have been published elsewhere.^25^ Because of the lack of randomization, there may be unmeasured confounders affecting the uptake in this study. Although we did not find major differences in the baseline characteristics between the groups, a randomized trial is ideal to measure health impact from such interventions. We did not directly observe handwashing behavior through structured observation, so it is possible that even though people had a better stocked handwashing station, this did not substantially impact their handwashing behavior at key times.

Soapy water promotion may increase habitual handwashing in resource-poor settings by addressing key handwashing agent barriers such as cost, sharing, and availability near a water source. Free provision of handwashing stations led to higher uptake. Providing a direct subsidy for a household level, durable product has implications on sustainability and how the users value their product. Delivering this intervention on a larger scale would require effective alternatives to free hardware delivery and specifically promoter-led behavior change strategies that are expensive to consistently supervise and to adequately revise to engage respondents over extended periods. Further studies are also needed to inform how soapy water will perform in other contexts, particularly in urban settings where resources are frequently shared among nonrelated families. Further research should determine approaches to effectively deliver this intervention at a larger scale and evaluate their effectiveness and health impact.

## Figures and Tables

**Table 1:** Baseline characteristics of households with children < 3 years old in rural Kishoreganj, Bangladesh, 2010

Characteristics	Control (*N* = 72)	Promotion-only (*N* = 120)	Promotion plus handwashing station (*N* = 103)	Promotion with handwashing station plus soapy water detergent refills (*N* = 90)
Female respondent, *n* (%)	50 (69)	117 (98)	94 (91)	83 (92)
Age of respondent (mean, SD)	31, 11.2	28, 6.7	31, 10.7	29, 10.1
Mother's education, *n* (%)				
None	16 (22)	35 (29)	39 (38)	27 (30)
Primary	34 (47)	49 (41)	41 (40)	29 (32)
Secondary	23 (32)	36 (30)	23 (22)	34 (38)
Father's education, *n* (%)				
None	26 (36)	50 (42)	51 (50)	42 (47)
Primary	29 (40)	46 (38)	25 (24)	21 (23)
Secondary	18 (25)	24 (20)	27 (26)	27 (30)
No. of household members eating from the same pot (mean, SD)	6.1, 2.6	5.2, 1.9	5.6, 1.7	5.7, 2.0
Primary handwashing place, *n* (%)				
Indoors	5 (6.9)	9 (7.6)	13 (11)	12 (11)
Outdoors	67 (91)	109 (92)	83 (72)	89 (83)
No fixed place	1 (1.7)	–	19 (17)	5 (4.7)
Had any soap at handwashing place, *n* (%)	4 (5.5)	15 (12)	5 (4.4)	17 (18)
Shallow tube well as water source, *n* (%)	60 (82)	118 (98)	94 (91)	89 (98)
Sanitation*, *n* (%)				
Improved latrine	25 (34)	26 (22)	37 (36)	30 (33)
Unimproved latrine	48 (66)	94 (78)	66 (64)	60 (67)

* Defined using World Health Organization/ United Nations International Children's Emergency Fund Joint Monitoring Program definition for sanitation.

**Table 2:** Location of handwashing place and handwashing demonstration at baseline and at 4-month follow-up, by treatment arm, Kishoreganj, Bangladesh, 2010

Primary handwashing place†	Control		Soapy water promotion-only		Soapy water promotion plus handwashing station		Soapy water promotion plus handwashing station plus free detergent refills	
	Baseline (*N* = 197)	Follow-up (*N* = 72)‡	Baseline (*N* = 145)	Follow-up (*N* = 120)	Baseline (*N* = 117)	Follow-up (*N* = 103)	Baseline (*N* = 106)	Follow-up (*N* = 90)
	%							
Indoors	11	6.9	8	2	12	19	11	23
Outdoors	83	92	92	94	72	81	84	77
< 10 steps from kitchen	31	45	31	28	54	55	50	71*§
< 10 steps from latrine	10	11	15	13	15	35*	29	46* §
Any soap/soapy water present	7.6	5.5	10	23	6.1	63	17	75
Soapy water present	–	–	–	18	–	60	–	71
Respondent used soap/soapy water in handwashing demonstration	67	64	70	84*	57	85*	65	91*
Clean hands observed	*N* = 121	*N* = 49	*N* = 134	*N* = 88	*N* = 113	*N* = 72	*N* = 98	*N* = 65
Mother	29	34	82	42	23	29	37	42
Child	40	52	47	58	77	71	63	58

* Significant differences from baseline, *P* < 0.05.

† Place where respondent reported washing hands most frequently.

‡ Smaller control group due to enrollment of initial control households in another intervention study.

§ Some primary handwashing places were < 10 steps from both the latrine and the kitchen indicating relatively small size of the compound.

**Table 3:** Differences in hygiene-related observations from baseline to follow-up for different treatment households compared with promotion-only, Kishoreganj 2010

Difference in different estimate (*P* value)	Station plus bottle (2) vs. promotion-only (1)	Station plus bottle plus refills (3) vs. promotion-only (1)	Station plus bottle (2) vs. station plus bottle plus refills (3)
Soap/soapy water and water together at primary handwashing station	47 (< 0.001)*	48 (< 0.001)*	1.6 (0.85)
Primary handwashing station			
< 10 steps from the latrine	24 (< 0.001)*	21 (0.03)	−3 (0.69)
< 10 steps from the kitchen	15 (0.09)	28 (0.003)*	13 (0.23)
Soap/soapy water near the kitchen	29 (0.001)*	37 (0.001)*	8.2 (0.37)
Soap/soapy water near the latrine	16 (0.018)	23 (0.014)*	6.7 (0.35)

The difference in difference estimate uses linear regression to estimate the differences in change in proportion from the baseline to follow-up across treatment groups.

* Significant following Bonferroni adjustment for multiple comparisons.
